# Effects of *Veratrum nigrum* expansion on soil microbial community structure in inner Mongolian mountain steppe

**DOI:** 10.3389/fmicb.2025.1662400

**Published:** 2025-10-28

**Authors:** Bo Pang, Hao Zhang, Tong Liu, Dianlin Yang, Hui Wang, Yanjun Zhang, Hongmei Liu, Haifang Zhang

**Affiliations:** Agro-Environmental Protection Institute, Ministry of Agriculture and Rural Affairs, Tianjin, China

**Keywords:** bacteria, expansion, fungi, microbial co-occurrence network, *Veratrum nigrum*

## Abstract

**Backgrounds:**

The expansion of poisonous weeds can significantly affect soil microbial communities and their ecosystem functions. In this regard, the poisonous weed *Veratrum nigrum* has impacted vast regions of Inner Mongolia, with potential impacts on the microbial community structure. However, the mechanism associated with this change remain unclear.

**Methods:**

In this study, we conducted a comparative analysis of four hazard levels (control CK, coverage = 0; light hazard L, coverage ≤20%; moderate hazard M, 20% < coverage ≤40%; and severe hazard S, coverage >40%) of *V. nigrum* in Inner Mongolia region, focusing on its effects on the soil physicochemical properties, microbial community structure, diversity, and co-occurrence patterns.

**Results:**

Results revealed that expansion of *V. nigrum* significantly altered the physicochemical properties and structure of the soil microbiota. The contents of available nitrogen, total nitrogen, total phosphorus, available phosphorus, and available potassium in the S treatment were significantly higher than those in the CK (*p* < 0.05), indicating that the diffusion of *V. nigrum* enhanced nutrient availability. Compared with CK, the fungal Chao1 and Shannon diversity increased significantly in the S treatment, whereas the abundance of oligotrophs (Basidiomycota) decreased significantly. In contrast, the Chao1 and Shannon indices of bacteria under S treatment showed no significant difference compared to CK. Correlation analyses indicated that soil fungal community composition was more sensitive to the changes in soil physicochemical properties caused by *V. nigrum* than bacterial community composition. Additionally, microbial co-occurrence network analyses revealed that the expansion of *V. nigrum* led to an increase in competitive microbial interactions. Structural equation modeling confirmed the influence of *V. nigrum* expansion and nutrient changes on the fungal community composition and diversity.

**Conclusion:**

This study reveals that *V. nigrum* expansion enhances nutrient availability and promotes the recovery of soil microbial diversity in degraded grasslands, particularly soil fungi, indicating that *V. nigrum* expansion serves as a self-protective mechanism for degraded grasslands. Thus, we aimed to challenge the conventional perspective that poisonous weeds are invariably harmful and to offer new insights into their role within degraded grassland ecosystems.

## 1 Introduction

The expansion of poisonous weed species has long been associated with the degradation of steppe ecosystems (Kaur et al., 2012; Sharma and Raghubanshi, 2009). In recent decades, these poisonous weeds have spread continuously (Wang et al., 2024), and serve as indicators of grassland degradation (Yang et al., 2024). Most studies have emphasized the negative effects of poisonous weeds, such as inhibiting forage growth, causing livestock poisoning, and resulting in economic loss. However, few studies have examined the potential positive effects of poisonous weeds on grassland ecology, including their role in enhancing nutrient availability, promoting microbial diversity, and alleviating the effects of overgrazing (Cheng et al., 2014). In fact, the spread of poisonous weeds is not the cause of grassland degradation but rather a consequence of their strong adaptive capacity (Zhang et al., 2020). Therefore, we aimed to challenge the conventional perspective that poisonous weeds are invariably harmful and to offer new insights into their role within degraded grassland ecosystems.

Soil microbiomes play key roles in ecosystems because of their intricate connections to biogeochemical cycling, energy flow, hydric retention regulation, and determination of soil fertility, agricultural productivity, and ecological resilience (Li et al., 2020; Hartmann and Six, 2023; Wall et al., 2015; Bardgett and van der Putten, 2014). In addition, soil microbial communities are widely recognized as significant indicators of ecosystem function because of their high sensitivity to soil conditions, which allows them to rapidly reflect the degree of soil degradation (Rodrigues et al., 2015). The expansion of toxic weeds can affect soil nutrient availability, resulting in significant changes in microbial diversity. Yang et al. (2024) found that the expansion of *Stellera chamaejasme* increased soil organic carbon, which contributed to the enrichment of Proteobacteria. Chen et al. (2023) found that *Ligularia virgaurea* influences microbial communities by altering soil pH and moisture content. In addition, changes in microbial community structure and diversity can further facilitate the colonization and spread of poisonous weeds. The expansion of *Cenchrus spinifex* increases the abundance of soil microorganisms associated with the nitrogen cycle, thereby modifying the soil nitrogen environment and promoting rapid growth (Ren et al., 2023). Similarly, *Ageratina adenophora* proliferation enhances the presence of beneficial bacteria, which induces a positive feedback effect on it (Sun et al., 2021). The expansion of these weeds may create favorable conditions for their growth and competition by modifying the soil environment, particularly through the alteration of soil microbial communities that are closely related to nutrient metabolism. Consequently, investigating the changes in microbial community structure and function resulting from the spread of poisonous plants is crucial. This research not only aids in understanding the mechanisms underlying underground invasion but also has significant implications for soil nutrient cycling in response to various disturbances.

*Veratrum nigrum* is a perennial poisonous herb that frequently serves as a companion species in natural grasslands, often displacing native plants and becoming a dominant species with increasing grassland degradation. According to survey data, the poisonous plant *V. nigrum* occupies an area of approximately 80,000 ha in the Hulunbeier grassland. Current research on *V. nigrum* has primarily focused on its morphological characteristics, geographical distribution, and medicinal properties; however, there are limited studies on the interactions between *V. nigrum*, soil environment, and soil microorganisms. Therefore, further investigation is essential to determine whether the expansion of *V. nigrum* can modify soil conditions, that further directly or indirectly influencing the structure of microbial communities to establish a self-promoting expansion mechanism.

Numerous studies have assessed the variations in microbial community succession resulting from poisonous weed type degraded grasslands (Jin et al., 2023). For instance, it has been observed that *A. adenophora* selectively accumulates bacteria, primarily *Clostridium* and *Enterobacter* (Chen et al., 2019). The diversity and abundance of soil microorganisms increased after the invasion of *Stellera chamaejasme* (Jin et al., 2018). *V. nigrum* is a perennial poisonous herb that frequently serves as a companion species in natural grasslands, often displacing native plants and becoming a dominant species with increasing grassland degradation. According to survey data, the poisonous plant *V. nigrum* occupies an area of approximately 80,000 ha in the Hulunbeier grassland. Current research on *V. nigrum* has primarily focused on its morphological characteristics, geographical distribution, and medicinal properties; however, there are limited studies on the interactions between *V. nigrum*, soil environment, and soil microorganisms. Therefore, further investigation is essential to determine whether the expansion of *V. nigrum* can modify soil conditions, that further directly or indirectly influencing the structure of microbial communities to establish a self-promoting expansion mechanism.

Bacteria and fungi are major constituents of the soil microbiome, and they respond differently to *V. nigrum* expansion due to variations in their life histories and physiological traits. Wang and Kuzyakov (2024) found that bacteria strongly outcompete fungi for simple substrates, while fungi take advantage of complex compounds. Yang et al. (2024) found that soil fungi are more sensitive to *S. chamaejasme* expansion than soil bacteria. In this study, we aimed to investigate differences in the diversity and community composition of soil fungi and bacteria during the expansion of *V. nigrum* succession. To achieve this, we selected four hazard levels based on *V. nigrum* coverage in the Inner Mongolia region of China: control (CK, coverage = 0), light hazard (L, coverage ≤20%), moderate hazard (M, 20% < coverage ≤40%), and severe hazard (S, coverage >40%). We hypothesized that (1) the spread of *V. nigrum* promotes the restoration of soil microbial diversity in degraded grasslands and (2) soil fungi are more sensitive than bacteria during the succession of *V. nigrum* expansion.

## 2 Materials and methods

### 2.1 Site descriptions

This study was conducted in Chen Barag Banner, located in Inner Mongolia, China. The geographical coordinates are 49°29’N and 119°21’E, with an average altitude of 687 m above sea level. The region is characterized by a semi-temperate and semi-arid continental climate within the mesothermal zone, with an annual mean temperature ranging from 0 to 3 °C and annual precipitation between 250 and 350 mm. The predominant grassland type in this area is mountain steppe, with dominant species including *Leymus chinensis*, *Achnatherum sibiricum*, *Poa attenuata*, *Thalictrum aquilegiifolium*, *Carex pediformis*, *Potential bifurca*, and *Sanguisorba officinalis*, among others.

### 2.2 Soil sampling and analyses

During the mid-July 2023 bloom period of *V. nigrum*, five transects were established along different directions in the natural grassland *V. nigrum* hazard area of Chenbalhu Banner, Inner Mongolia, in which four 100*100 m sample sites were established according to the gradient of *V. nigrum* hazards, respectively, as control CK (no *V. nigrum*), light hazard L (cover ≤ 20%), medium hazard M (20% < cover ≤ 40%), and severe hazard S (cover > 40%), and three quadrats (1 m × 1 m) were installed in each sample site. Each sample site was separated by a minimum distance of 1,000 m, and each quadrat was separated by a minimum distance of 10 m. A mixture of three replicate soil samples (5 cm in diameter and 20 cm in depth) was collected in each quadrat according to the S-shape sampling pattern. The soil was thoroughly mixed and collected using the quartering method. The soil samples were then sieved through a 2-mm screen, transported to the laboratory in sterile plastic containers, and packed with dry ice. A portion of the soil was air-dried and passed through a 100-mesh sieve for physicochemical analysis, while the remaining portion was stored at −80 °C for microbial analyses.

### 2.3 Analysis of physicochemical properties of soil

A Portable Multi-Parameter Meter (DZB-712, Shanghai, China) was used to measure the soil pH, and a flow injection auto-analyzer (AA3, Seal Co., Germany) was used to determine the concentrations of ammonium (NH_4_^+^ − N) and nitrate (NO_3_^−^−N) in the soil. The total nitrogen (TN) concentrations were assessed using the Kjeldahl digestion method. Soil organic matter (SOM) content was measured using the potassium dichromate external heating method, and total phosphorus (TP) in the soil was quantified using molybdenum-antimony resistance colorimetry. Sodium bicarbonate was used to extract available phosphorus (AP) from the soil, which was then quantified using the molybdenum blue method. Available potassium (AK) in the soil was extracted using ammonium acetate and subsequently quantified using Atomic Absorption Spectroscopy (Bao, 2000).

### 2.4 Analysis of microbial community by MiSeq sequencing of 16S rRNA and internal transcribed spacer gene amplicons

Total microbial DNA from the mixed soil samples was extracted using the E. Z. N. A soil DNA extraction kit (Omega, United States) following the manufacturer’s instructions. The concentration and purity of DNA were assessed using a NanoDrop 2000 spectrophotometer, whereas its integrity was determined using 1% agarose gel electrophoresis. The V3-V4 hypervariable regions of the bacterial 16S rRNA gene were amplified using the primers 338F (5′-ACTCCTACGGGAGGCAGCAG-3′) and 806R (5′-GGACTACHVGGGTWTCTAAT-3′) (Claesson et al., 2009; Mori et al., 2014). Primers ITS1F (5′-CTTGGTCATTTAGAGGAAGTAA-3′) and ITS2R (5′-GCTGCGTTCTTCATCGATGC-3′) were used for the fungal PCR (Gardes and Bruns, 1993). Raw high-throughput sequencing data were subjected to an initial assessment for sequence quality. Barcodes were used to separate the samples into libraries, and the barcode sequences were removed. Sequences that were longer than 200 bp and had a mean quality score of at least 20 were selected for further analysis. The DADA2 method was used for primer removal, mass filtration, denoising, splicing, and removal of chimeric sequences, for which sequences with ≥97% similarity were assigned to the same operational taxonomic unit (OTU). All PCR were performed in a 20 μL reaction volume, which included 10 μL of 2 × Pro Taq, 0.8 μL each of 5 μM forward and reverse primers, 10 ng/μL of DNA, and distilled deionized water to bring the total volume to 20 μL. The thermal cycling conditions for bacteria and fungi were as follows: for bacteria, denaturation at 95 °C for 3 min, followed by 29 cycles of 95 °C for 30 s, 53 °C for 30 s, 72 °C for 45 s, and a final extension at 72 °C for 10 min; for fungi, denaturation at 95 °C for 3 min, followed by 35 cycles of 95 °C for 30 s, 55 °C for 30 s, 72 °C for 45 s, and a final extension at 72 °C for 10 min. Each sample was subjected to three PCR cycles and was mixed after amplification.

### 2.5 Statistical analyses

The data were analyzed for physicochemical properties and microbial *α*-diversity (Shannon and Chao1 indices) using Microsoft Excel 2011. A one-way analysis of variance was conducted using the R package (version 4.4.1). Dunn’s test was performed for *post hoc* analysis. Beta (*β*) diversity was assessed through principal coordinates analysis (PCoA) based on the Bray-Curtis distance matrix of normalized operational taxonomic unit (OTU) data in the R package (version 4.4.1). To evaluate significant differences in community structure across various treatments at the phylum level, permutational multivariate analysis of variance (PERMANOVA) was used. This algorithm was performed using the adonis function provided in the R vegan package. To evaluate the correlations between microbial composition and soil physicochemical properties, we performed Spearman’s correlation analysis between the composition of dominant species, based on a relative abundance >1% threshold at the phylum level, and various soil physicochemical parameters. Additionally, we conducted microbial co-occurrence network analysis to assess the effects of different treatments on microbial interactions and the complexity of soil fungal and bacterial communities during the expansion process. OTUs with a relative abundance of <0.01% were deleted to minimize the presence of rare OTUs in the data set. Spearman’s correlation coefficient was used to evaluate the correlation between OTUs, adjusted for Benjamini and Hochberg’s (2018) false discovery rate. Correlation coefficients >0.8, along with corresponding *p*-values <0.01, were considered statistically significant and included in the network generation. To evaluate the microbial associations and network complexity across various treatments, different network topological properties were calculated. These properties included the total number of nodes and edges, positive and negative connections, average clustering coefficient, average path distance, modularity, and average degree. Bacterial and fungal co-occurrence networks were visualized using Gephi (version 0.10.1) (Bastian et al., 2009). To examine the influence of soil characteristics on bacterial and fungal diversity and community composition under various levels of damage to *V. nigrum*, structural equation modeling (SEM) was used, incorporating regression analysis and prior experience. The final model was refined by systematically eliminating non-significant pathways from the previous model, based on the aforementioned indices. The adequacy of the model was evaluated using several metrics, including the chi-square (*χ*^2^) test, comparative fit index, goodness-of-fit index, and root mean square error of approximation. SEM analysis was conducted using the AMOS 22.0 software program (AMOS Development Corporation).

## 3 Results

### 3.1 Soil properties

The expansion of *V. nigrum* resulted in a decrease in soil pH compared with CK, with the soil pH in the L, M, and S treatments declining by 0.49, 0.97, and 3.73%, respectively. The SOM content exhibited an increase, followed by a decrease, with the L and M treatments showing significantly higher levels than CK (*p* < 0.05), whereas no significant difference was observed between the S treatment and CK. Additionally, the NH_4_^+^ − N and TP contents were significantly higher in the M and S treatments than in the CK and L treatments (*p* < 0.05). The contents of NO_3_^−^−N, TN, AP, and AK increased with the degree of hazard, indicating that the levels of NO_3_^−^−N, TN, AP, and AK in the L, M, and S treatments were significantly higher than those in the CK (*p* < 0.05).

### 3.2 Microbial diversity and community composition

**Figure 1:**
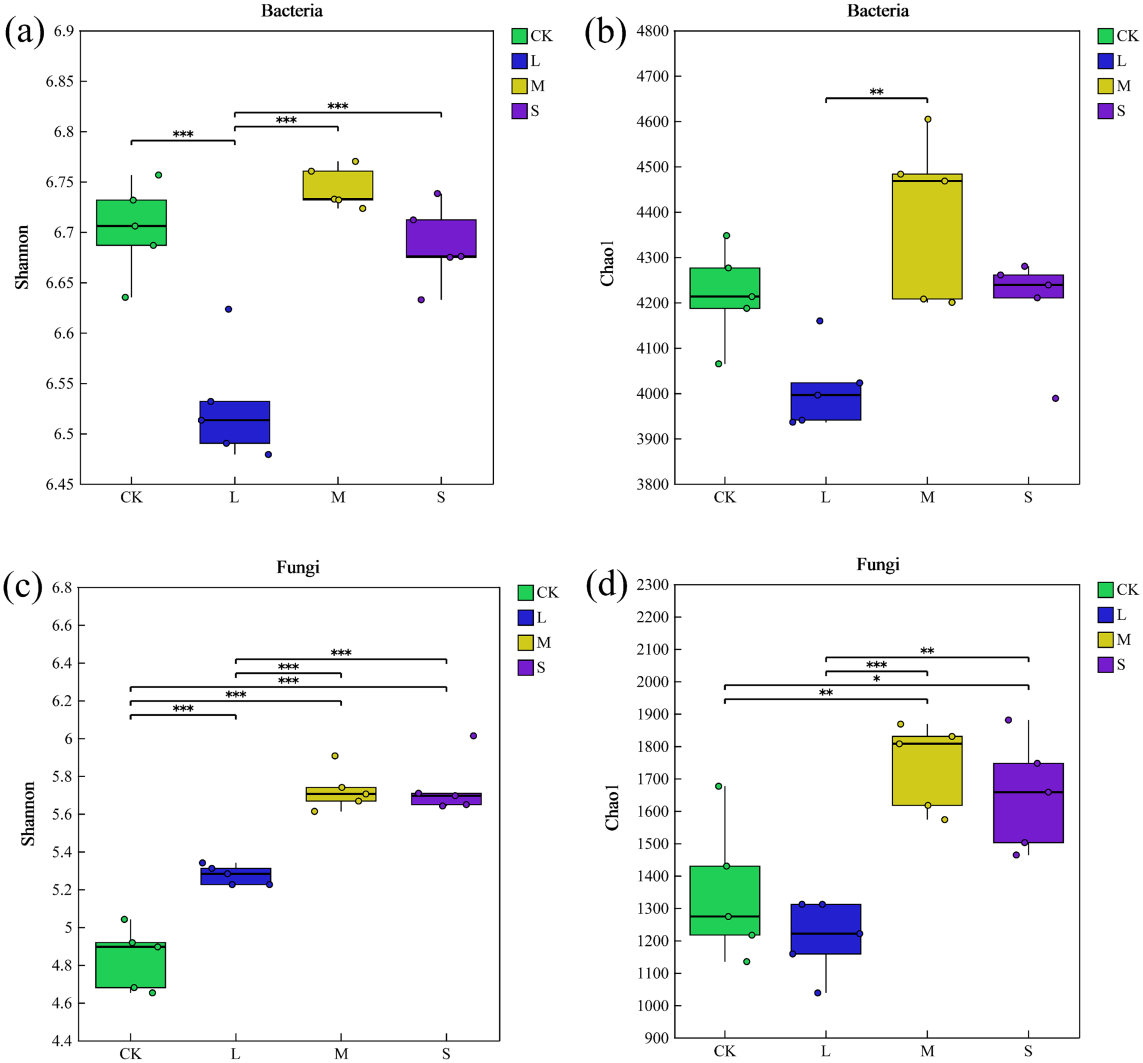
Alpha diversity (Shannon and Chao1 indices) of bacterial **(a,b)** and fungal **(c,d)** communities. Symbols in the plots indicate significant differences (*t*-test, *p* < 0.05) between both groups (“***” indicates *p* < 0.001, “**” indicates *p* < 0.01, “*” indicates *p* < 0.05).

The Chao1 and Shannon indices for fungi increased by 23 and 19%, respectively, under the S treatment, indicating a significant enhancement in soil fungal richness and diversity compared with CK (Figures 1c and d). However, there was no significant difference in bacterial alpha diversity under S treatment compared to CK (*p* > 0.05, Figures 1a and b).

**Figure 2:**
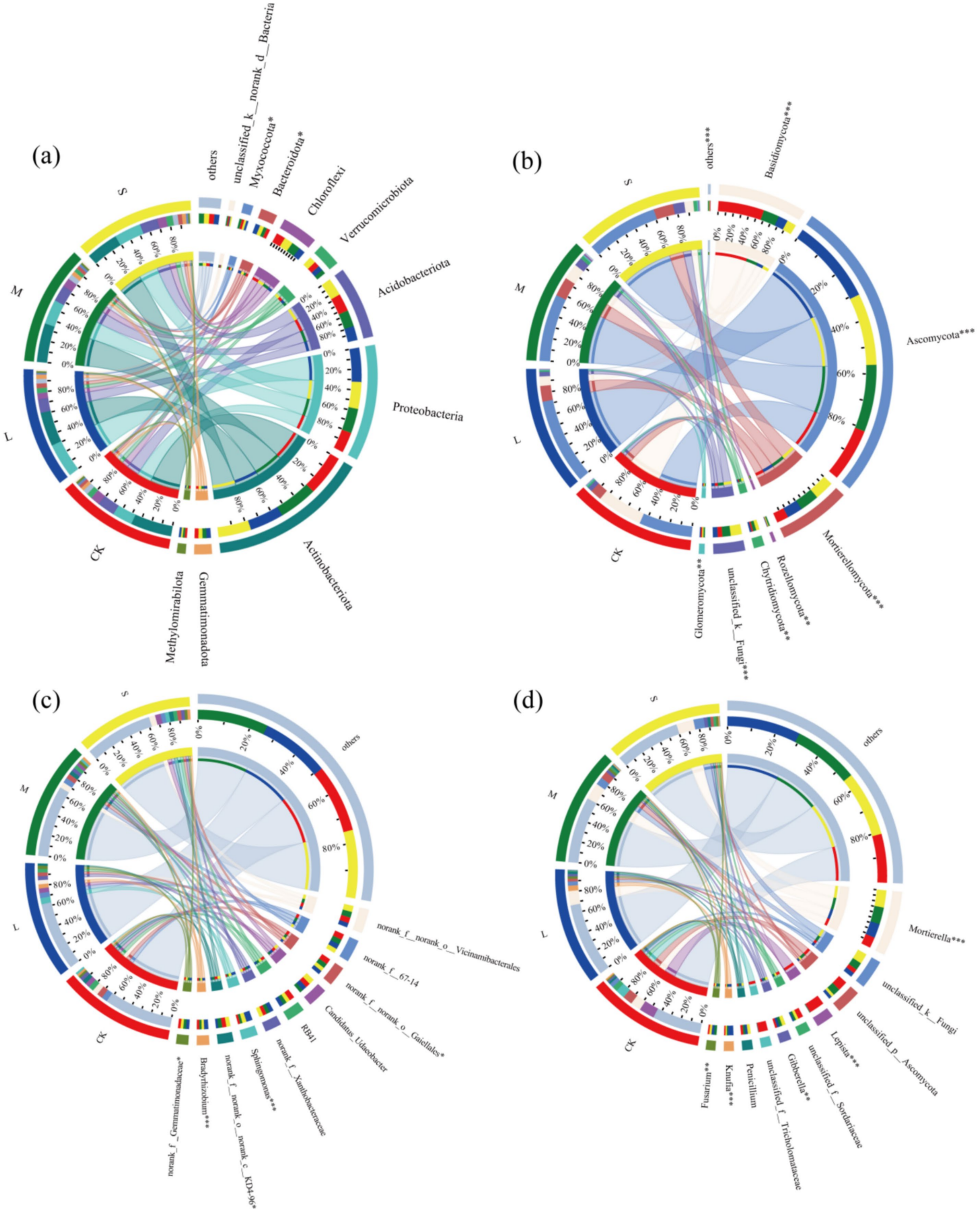
Relative abundance of dominant phyla (>1%) in **(a)** bacterial and **(b)** fungal communities. Relative abundance of dominant genera (>10) in **(c)** bacterial and **(d)** fungal communities.

Chord diagrams indicated that the dominant bacterial phyla were Actinobacteriota, Proteobacteria, Acidobacteriota, and Chloroflexi (Figure 2a). The dominant soil bacterial species remained consistent at the phylum level across the different treatments, suggesting that the expansion of *V. nigrum* did not alter the dominant phyla of soil bacteria. At the genus level, the dominant taxa included *norank_f__norank_o__Vicinamibacterales*, *norank_f__67–14*, *norank_f__norank_o__Gaiellales*, among others (Figure 2c). Notably, the relative abundance of *Sphingomonas* in the S treatment was significantly higher than in the CK.

The dominant fungal phyla were Ascomycota, Basidiomycota, and Mortierellomycota (Figure 2b). In soil fungi, the L, M and S treatments led to an increase in the relative abundance of Ascomycota and Mortierellomycota, whereas the relative abundance of Basidiomycota decreased (*p* < 0.05). In S-treated soil, the relative abundances of Ascomycota and Mortierellomycota increased by 35.7 and 71.2%, respectively, whereas that of Basidiomycota decreased by 80.3% (Figure 2b) At the genus level, *Mortierella*, *Lepista*, *Gibberella* and other genera were dominant (Figure 2d). Notably, the relative abundances of *Mortierella*, *Knufia*, *Fusarium* and *Gibberella* in the S treatment were significantly higher than those in the CK treatment, whereas those of *Lepista* was significantly lower than those in the CK (Figure 2d). In summary, our findings indicate that varying degrees of *V. nigrum* harm significantly influence the composition of soil microbial communities.

**Figure 3:**
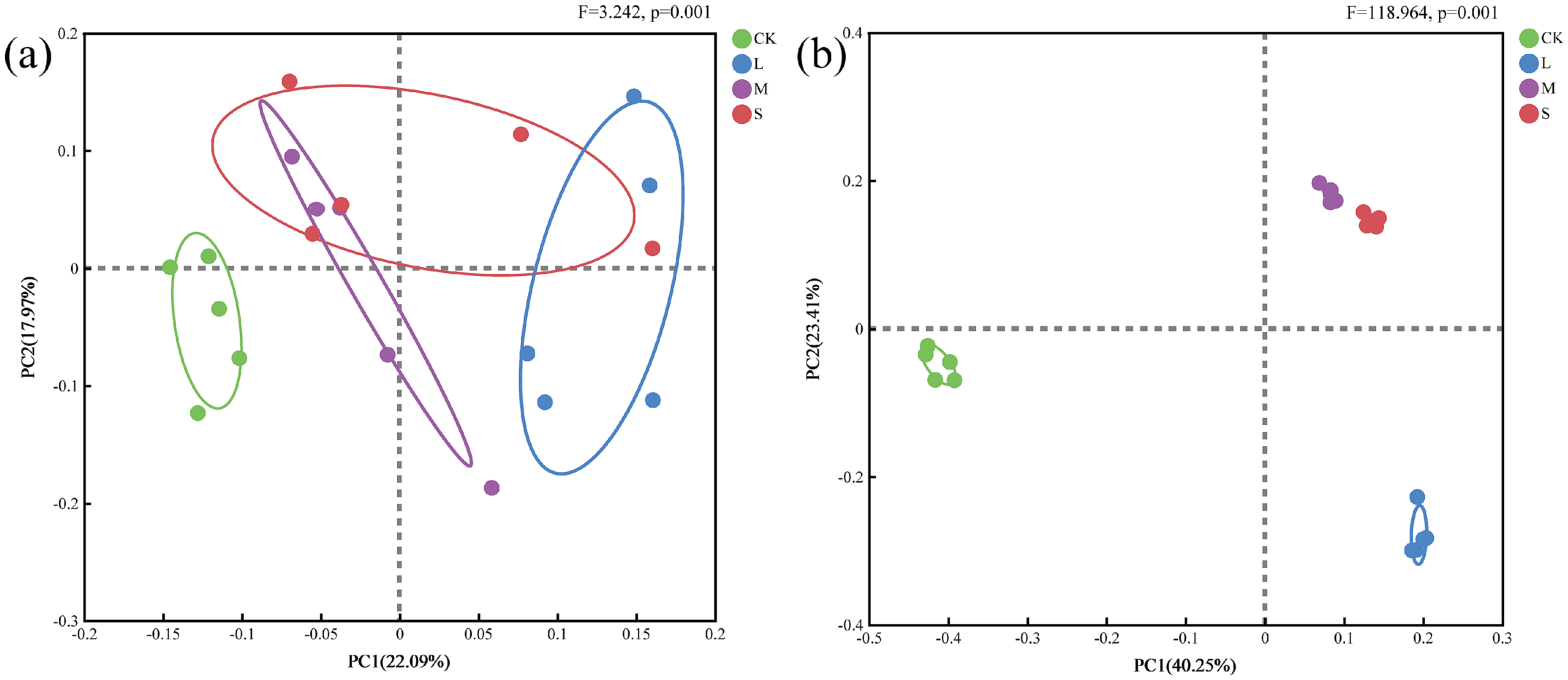
**(a)** Bacterial and **(b)** fungal composition structures (at the OTU level) determined using PCoA.

The principal coordinate analysis (PCoA) clearly showed that CK was distinct from the other treatments in terms of the soil bacterial community, with PCo1 accounting for 22.09% of the total variation (Figure 3a). In contrast, the bacterial communities in the L-, M-, and S-treatment groups were highly similar. Furthermore, the PCoA revealed significant differences in soil fungi across varying hazard levels, with PCo1 explaining 40.25% of the total variation (Figure 3b). According to PERMANOVA, a significant difference was observed in the beta diversity of the bacterial communities (*F* = 3.242, *p* = 0.001) across different hazard levels, as well as a significant difference in the beta diversity of the fungal communities (*F* = 118.964, *p* = 0.001).

### 3.3 Co-occurrence of bacteria and fungi

**Figure 4:**
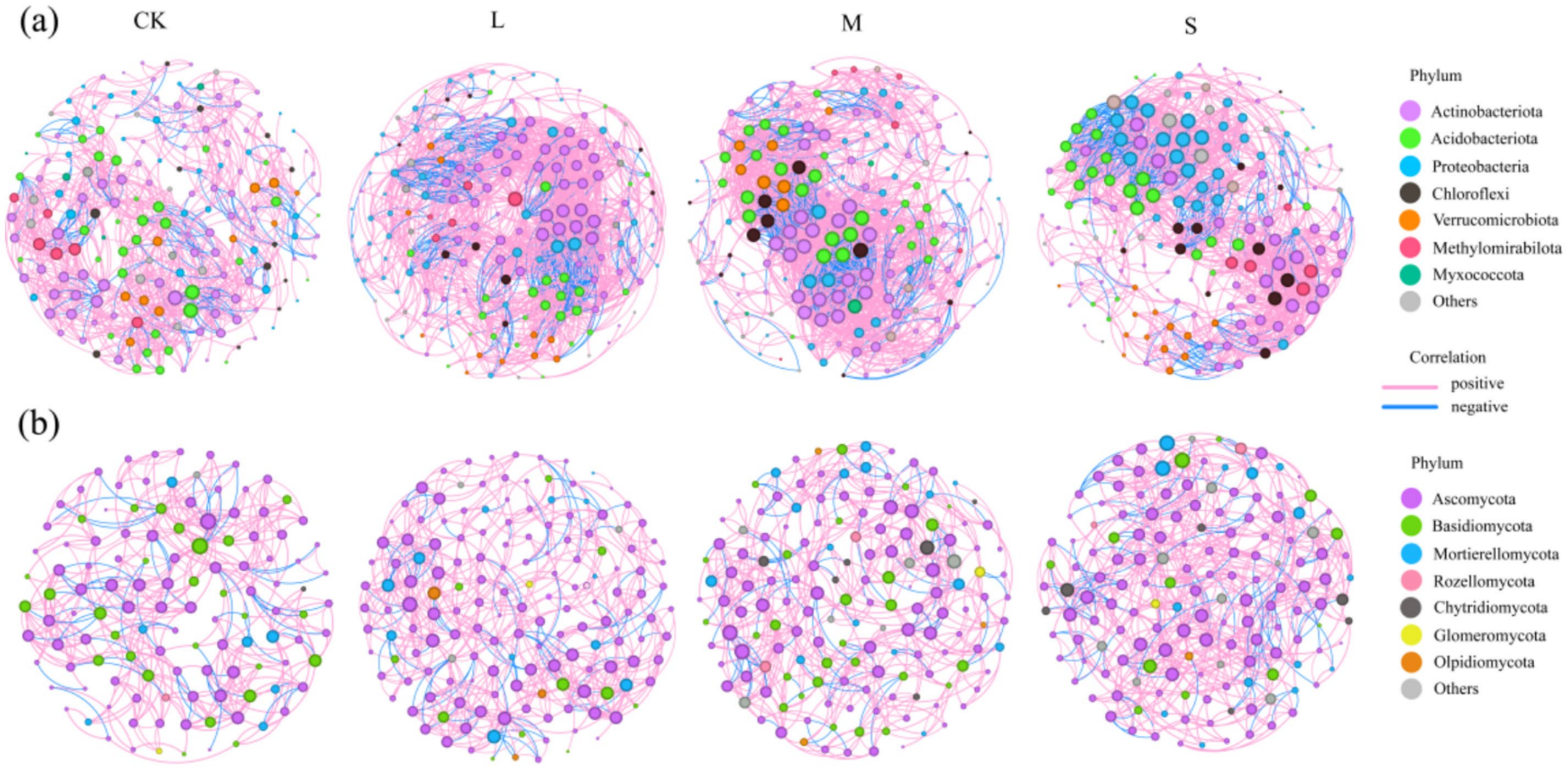
Network analysis revealing the co-occurrence pattern between **(a)** bacterial and **(b)** fungal OTUs in soil treated with CK, L, M, and S. Colored nodes represent the corresponding OTUs assigned to major phyla. The red and blue lines represent positive linear relationship (*p* > 1) and negative linear relationship (*p* < 1) and relationships, respectively. The size of each node is proportional to the number of connections (degree).

To determine the general effects of varying hazard levels on soil microbiota associations, co-occurrence networks of bacteria and fungi were generated separately across different hazard gradients and the associated topological properties were calculated (Figure 4; Supplementary Table S1). Distinct patterns of co-occurrence were observed in the soil bacteria and fungi in response to *V. nigrum* expansion. Compared with CK, the total number of links in the bacterial and fungal networks increased by 49.7 and 49.9%, respectively, in the S treatment. The average degree of the bacterial co-occurrence network increased by 84.2% with the S treatment, indicating that the expansion of *V. nigrum* enhanced the complexity of the bacterial community, whereas the fungal network did not exhibit significant changes (Figure 4; Supplementary Table S1). Furthermore, the number of negative links in the bacterial and fungal networks increased by 10.53 and 8.82%, respectively, during the expansion of *V. nigrum* (Supplementary Table S1).

### 3.4 Environmental factors associated with bacterial and fungal community in response to *Veratrum nigrum*

**Figure 5:**
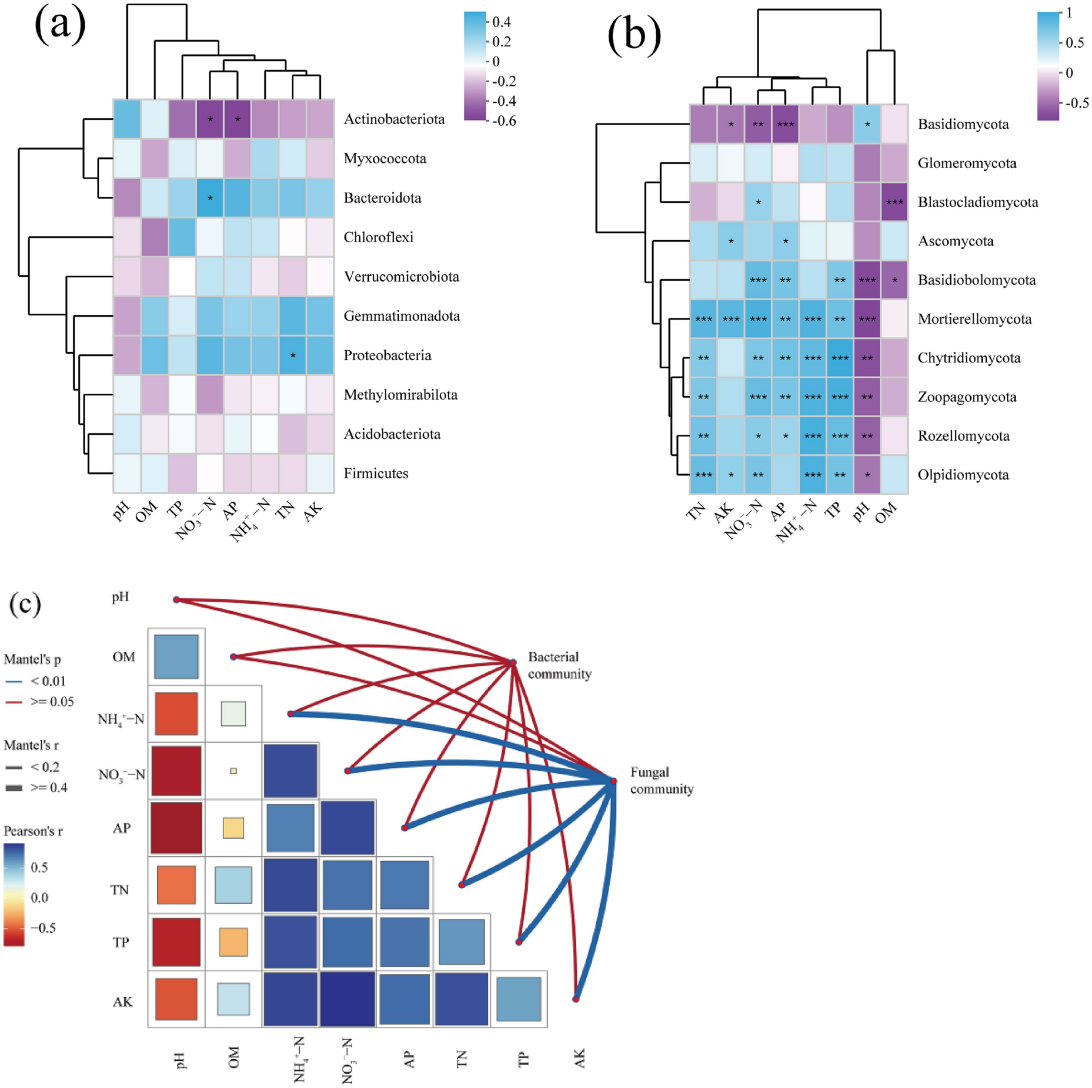
**(a,b)** Spearman’s correlation analysis between the relative abundances of bacteria and fungi at the phylum level and various soil physicochemical properties. *, **, and *** denote statistically significant correlations at the 0.05, 0.01, and 0.001 significance levels, respectively. **(c)** Mantel test between microbial communities (at the phylum level) and various soil physicochemical properties.

The Mantel test results indicated that environmental factors exerted a strong influence on fungal communities resulted from *V. nigrum* expansion, whereas there was no significant effect on bacterial communities at the OTUs level. (Figure 5c). A significant positive correlation was observed between fungal community composition and soil N, P, and AK content at different hazard levels (*p* < 0.05, Figure 5c). Spearman’s correlation analysis revealed that soil pH, TN, TP, NO_3_^−^−N, and NH_4_^+^ − N content mainly influenced the relative abundance of fungal phyla (Figure 5b). Specifically, the abundance of *Mortierellomycota* was positively correlated with soil TN, TP, NO_3_^−^−N, and NH_4_^+^ − N content (*p* < 0.05), and negatively correlated with soil pH (*p* < 0.05). The abundance of *Basidiobolomycota* was negatively correlated with SOM content (*p* < 0.05), whereas the abundance of *Ascomycota* was negatively correlated with soil AP and AK contents (*p* < 0.05, Figure 5b). In contrast, the relative abundance of the dominant bacterial phylum was less affected by the soil physicochemical properties than that of fungi (Figure 5a). Notably, the abundance of *Actinobacteriota* was negatively correlated with soil AP and NO_3_^−^−N content (*p* < 0.05), whereas the abundance of *Bacteroidota* was positively correlated with soil NO_3_^−^−N content (*p* < 0.05).

**Figure 6:**
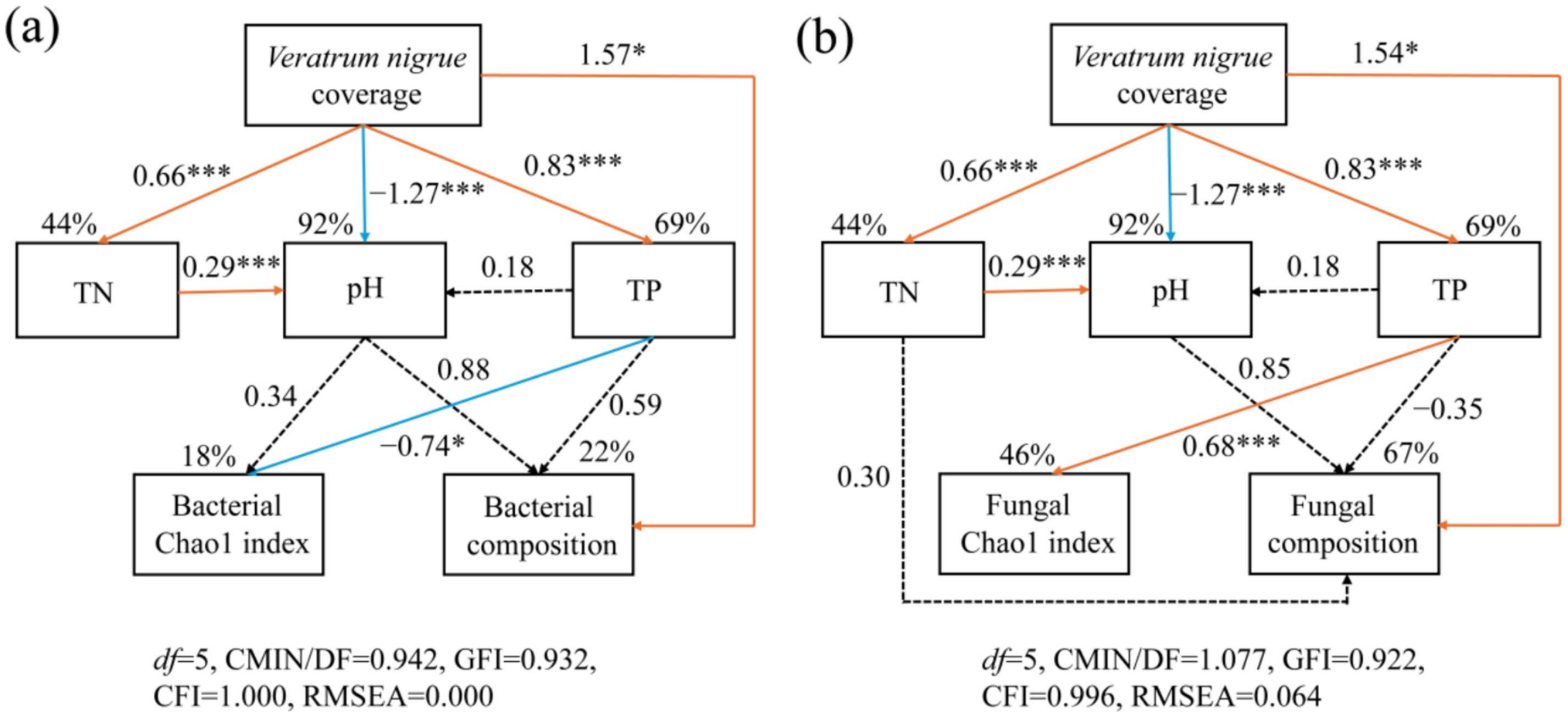
SEM outlining the influence of the expansion of *V. nigrum* andsoil properties (TN, pH, TP) on the contribution of bacterial **(a)** and fungal **(b)** Chao1 indices and community composition. The single-headed arrows indicate the positive and negative relationships, respectively. Solid orange lines denote positive relationships, whereas solid blue lines signify negative relationships at significance levels of *p < 0.05, **p < 0.01, and ***p < 0.001. Dotted lines denote non-significant paths. The numbers adjacent to the arrows correspond to standardized by the path coefficients, which indicate the effect size of the relationships. The R^2^ value reflects the variance in biomarkers explained by the model. For the composite variable of microbial composition, we integrated the bacterial composition (Axis 1 of PcoA analysis based on OTU abundance) and the fungal composition (Axis 1 of the PcoA analysis based on OTU abundance).

**Figure 7:**
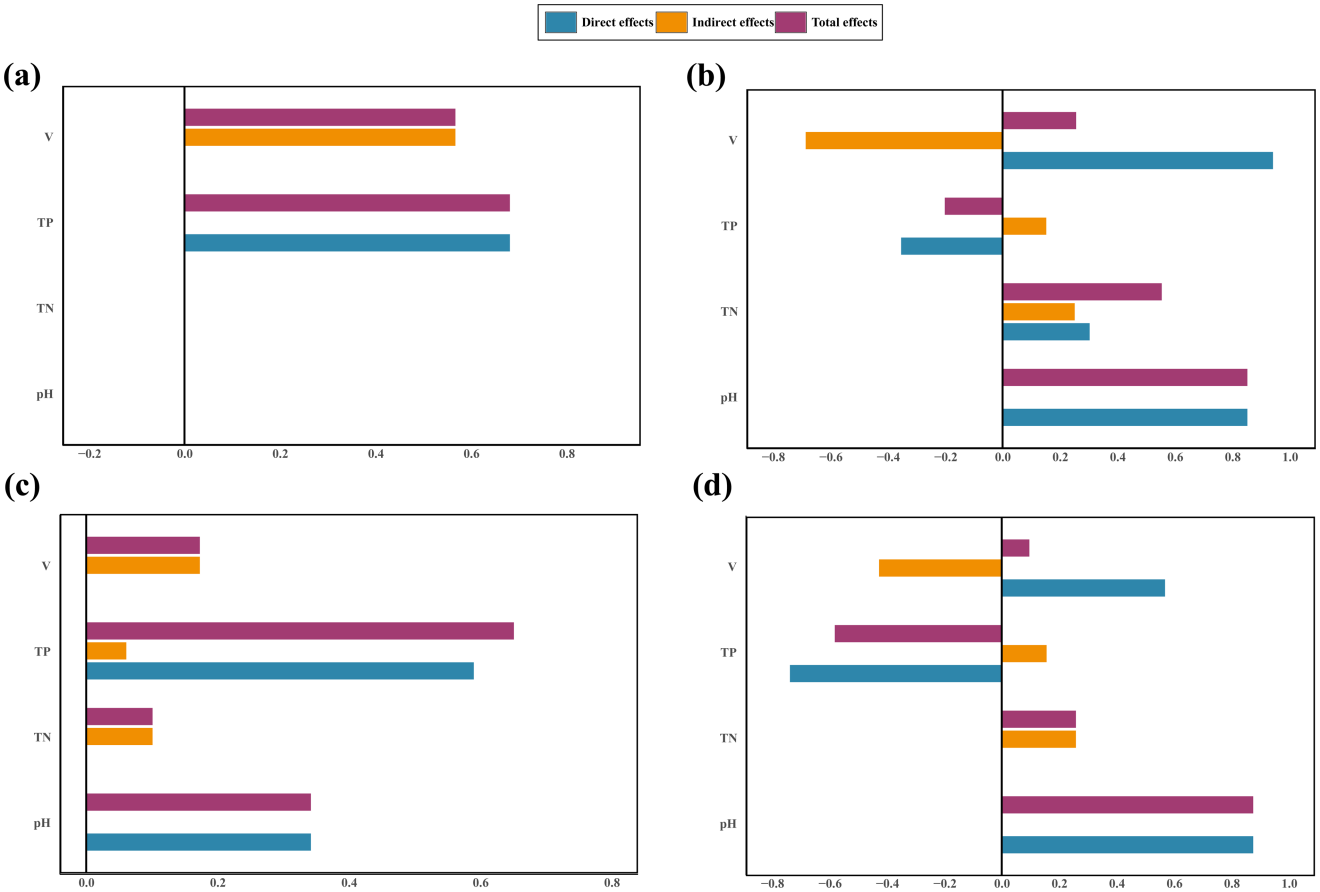
Standardized direct, indirect, and total effects of the Chao1 index and community composition on fungi **(a,b)** and bacteria **(c,d)** in SEM paths. V, *V. nigrum* coverage.

The relationship between the soil properties, microbiome, and expansion of*V. nigrum* was elucidated using Structure equation models (SEM) analysis (Figure 6). The expansion of *V. nigrum* had a significant positive effect on TN and TP (*p* < 0.05) and a significant negative effect on pH (*p <* 0.05). The expansion of *V. nigrum* had direct effects on fungal community composition, and soil TP content showing the most significant direct effect on the fungal Chao1 index (Figure 7). Furthermore, soil properties had a limited effect on bacterial communities (*p* > 0.05) but had a more pronounced effect on fungi than on bacteria. TP had a significant positive effect on fungal diversity (*p* < 0.05).

The SEM model accounted for 46 and 67% of the variance in the soil fungal Chao1 index and community composition, respectively (Figure 6b).

## 4 Discussion

### 4.1 Soil physicochemical properties shift due to the expansion of *Veratrum nigrum*

Poisonous weeds can induce ecological changes in the soil by releasing allelopathic substances (Ain et al., 2023; Zhu et al., 2020). In this study, the soil pH significantly decreased as the expansion of *V. nigrum* increased. This phenomenon may be related to the secretion of various secondary metabolites, such as organic acids by the root system of *V. nigrum* (Jia et al., 2021). Organic acid compounds have been shown to lower the soil pH, which may directly contribute to the observed decrease in pH (Rukshana et al., 2012). Notably, the available nitrogen content can significantly increase when the soil pH falls within the acidic range. This increase is attributed to enhanced microbial activity at this pH level, which promotes more efficient nutrient transformation and availability in the soil, ensuring that these nutrients are readily accessible for plant uptake (Li et al., 2020). The results of the present study also confirmed that the available nitrogen content in the S treatment was higher than that in CK (Table 1). Furthermore, *V. nigrum* is a C_4_ plant and its aboveground litter exhibits higher production (Cheng et al., 2010). This litter material enhances nitrogen availability by influencing soil microbial biomass and activity (Sun et al., 2009; Almagro et al., 2021). This effect may also contribute to the increase in the available nitrogen content in the soil.

**Table 1:** Physicochemical characteristics of soil.

	CK	L	M	S
pH	6.17 ± 0.03a	6.14 ± 0.02a	6.11 ± 0.04b	5.94 ± 0.02c
SOM (g·kg^−1^)	50.86 ± 9.50b	63.17 ± 5.26a	62.15 ± 6.58a	45.03 ± 3.68b
NH_4_^+^ − N (mg·kg^−1^)	12.04 ± 0.97c	17.45 ± 2.11b	22.89 ± 5.01a	21.02 ± 3.96a
NO_3_^−^−N (mg·kg^−1^)	13.51 ± 1.47c	17.82 ± 0.25b	17.88 ± 0.405b	19.81 ± 0.71a
AP (mg·kg^−1^)	11.39 ± 0.92c	14.96 ± 1.64b	15.08 ± 2.98b	18.31 ± 0.68a
TN (g·kg^−1^)	4.56 ± 0.41b	5.77 ± 0.32a	6.59 ± 1.31a	6.02 ± 0.79a
TP (g·kg^−1^)	0.61 ± 0.07b	0.62 ± 0.03b	0.67 ± 0.03a	0.68 ± 0.02a
AK (mg·kg^−1^)	408.27 ± 24.77b	604.17 ± 20.79a	610.43 ± 30.64a	610.33 ± 24.97a

SOM, soil organic matter; NH_4_^+^ − N, ammonium nitrogen; NO_3_^−^−N, nitrate nitrogen; AP, available phosphorus; TN, total nitrogen; TP, total phosphorus; AK, available potassium. Different lowercase letters denote significant differences between levels of coverage (*p* < 0.05; ANOVA).

Previous studies have shown that invasive poisonous plants have a strong ability to maintain soil nutrients (Fan et al., 2010; Chen et al., 2009). For instance, species such as *S. chamaejasme* L., *Lantana camara* L., and *Phytolacca americana* L. have been observed to enhance the concentrations of available elements, such as nitrogen and phosphorus, in the soil. This results in higher nutrient use efficiency compared with that of native species, thereby alleviating nutrient stress in the environment (Cheng et al., 2022; Sharma and Raghubanshi, 2009; Meng et al., 2024). This study found that soil TP and AP contents were significantly higher under the S treatment than under CK. This indicates that the spread of *V. nigrum* enhances the availability of soil phosphorus, which aligns with the findings of Spiegelberger et al. (2006). In addition, the roots of *V. nigrum* secrete substantial amounts of phenolic compounds, specifically caffeic and chlorogenic acids (Wang, 2007). Batish et al. (2002) reported a positive correlation between phenolic compounds and AK; therefore, the observed increase in soil AK content with the expansion of *V. nigrum* can be attributed to the influence of phenolics. In summary, the contributions of leaf litter and root secretions to the soil environment, along with changes in soil physicochemical properties and nutrient composition, particularly the enhancement of soil fertility, create a favorable environment for the successful invasion, growth, and expansion of *V. nigrum* (Wang, 2007).

### 4.2 Soil microbial diversity and community shifts due to expansion of *Veratrum nigrum*

Microbial community diversity and composition exhibited varying responses to different hazard levels of *V. nigrum* (Figures. 1, 2). Specifically, the diversity of soil fungi shifted significantly during the expansion of *V. nigrum*, whereas that of the bacteria remained relatively unchanged (Figure 1). This indicates that the fungal diversity index is more sensitive to the expansion of *V. nigrum* than to that of bacteria, which aligns with the second hypothesis. Furthermore, we observed that the Chao1 and Shannon diversity indices for fungi were higher in S-treated soils than in the CK (Figure 1). This trend corresponds to fluctuations in soil nutrient levels, suggesting that soil nutrients may mediate changes in fungal diversity (Liu et al., 2022; Yang et al., 2022), which was further supported by the SEM results of this study. Notably, the Chao1 index accounts for species richness and reflects variation in rare species (Chao and Lee, 1992). Thus, the presence of rare fungal species was significantly correlated with *V. nigrum* expansion (*p* > 0.05).

Soil fungi play a crucial role as decomposers and plant symbionts in soil ecosystems (Tan et al., 2021). Across all treatments, Ascomycota, Basidiomycota and Mortierellomycota were the dominant fungal phyla (Figure 4), which was consistent with previous studies on the invasion of poisonous weeds (He et al., 2019; Jin et al., 2018). In the fungal community, the relative abundance of Ascomycota and Mortierellomycota increased in the L, M, and S treatments compared with that in CK, whereas the relative abundance of Basidiomycota decreased. The observed changes in the relative abundances of these fungal phyla can be attributed to variations in soil nutrients (Sui et al., 2022), which confirms that the composition of the soil fungal community is particularly sensitive to changes in the soil environment (Figure 5). In addition, secondary metabolites produced by *V. nigrum* may reshape the fungal community owing to substrate preferences (Zhalnina et al., 2018). For instance, Chen et al. (2020) found that cyanide produced by neighboring plants contributes to ethylene release in peanut roots, thereby mediating the reconstruction of rhizosphere microbial communities. Root growth and chemical traits also influence the composition of fungal communities (Wan et al., 2021).

The LEfSe analysis revealed that bacterial biomarkers in the light-hazard site were predominantly Proteobacteria, whereas fungal biomarkers were primarily Ascomycota (Supplementary Figure S1). Proteobacteria play a crucial role in the biological cycling of essential mineral nutrients in the soil (Kim et al., 2021). Actinobacteria are involved in organic matter cycling and facilitate the degradation of plant and animal residues in the soil (Javed et al., 2021). The expansion of *V. nigrum* increased SOC, which may explain why Proteobacteria and Actinobacteria were identified as biomarkers of light hazard areas (Schneider et al., 2012). In addition, fungal biomarkers in the CK treatment were predominantly Basidiomycota, indicating a decline in substrate quality in the absence of *V. nigrum* expansion, with only recalcitrant carbon compounds (e.g., lignin) remaining available (Fanin and Bertrand, 2016; Voříšková and Baldrian, 2013). This is attributed to their capacity to synthesize enzymes necessary for the degradation of complex polymers (Baldrian, 2008). In contrast, the fungal biomarker in the heavy hazard areas of *V. nigrum* was primarily Blastocladiomycota. This can be attributed to root turnover, which generates a substantial amount of decomposed organic matter. This provided additional carbon sources for the vital activities of Blastocladiomycota (Gleason et al., 2018). Furthermore, the positive correlation between SOM and Blastocladiomycota supported this conclusion (Figure 6). In summary, the expansion of *V. nigrum* ultimately contributes to the restoration of microbial diversity in degraded grassland soils, despite its varying effects on the diversity and composition of the bacterial and fungal communities.

### 4.3 Expansion of *Veratrum nigrum* increases complexity of microbial co-occurrence networks

Microorganisms do not exist in isolation, rather, they form complex networks of ecological interactions that play a crucial role in maintaining soil functions such as plant nutrient acquisition and soil formation (Faust and Raes, 2012). We used microbial co-occurrence network analysis and examined network topology features to explore interactions among microbial communities. Our results indicated that the average clustering coefficients of the bacterial and fungal networks (approximately 0.602 to 0.701) were significantly higher than those of their corresponding random networks (approximately 0.01 to 0.04) (Supplementary Table S1). All modularity index values were >0.4, indicating a typical modular structure (Newman, 2006) and strong resistance to environmental changes, which is consistent with the findings of Bai et al. (2017). In this study, we found that soil bacteria and fungi were positively associated throughout the dispersal process (Figure 6), which was consistent with the findings of Yang et al. (2022). We speculate that synergistic relationships among soil microorganisms are vital in meadow ecosystems, and the mechanisms of these interactions vary across different dispersal periods. During the period without *V. nigrum* expansion, different species primarily cooperate to withstand harsh environmental conditions and maintain their survival. However, the increased negative correlation between the bacterial and fungal networks in the S treatment indicated an increase in the number of microorganisms competing for mutualistic interactions in the soil after the expansion of *V. nigrum*. This phenomenon can be attributed to the increased nutrient availability after the expansion of *V. nigrum*, which led to a greater number of species with similar ecological niches adapting to environmental changes (Gong et al., 2024). Hernandez et al. (2021) found that low environmental stress enhances microbial competitive interactions. Conversely, a previous study indicated that increased resources lead to an increase in shared ecological niches; thus, the positive correlation between different species remains dominant (Wei et al., 2020). Furthermore, we observed that the complexity of the bacterial co-occurrence network increased during the expansion of *V. nigrum*, although bacterial diversity did not change (Figure 4). This finding was consistent with the results reported by Yang et al. (2023), who suggested that changes in soil microbial diversity do not always correlate with alterations in the microbial network (Yang et al., 2023).

### 4.4 Relationships among expansion of *Veratrum nigrum*, soil physicochemical characteristics, microbiota

SEM results indicated that the explanatory models for fungal Chao1 and community composition accounted for 46 and 67% of the variance, respectively (Figure 6). In contrast, the models for bacterial Chao1 and community composition explained only 18 and 22% of the variance, respectively (Figure 6). This suggests that the cover gradient of *V. nigrum* and the measured soil physicochemical properties inadequately accounted for the changes in the bacterial community structure. Furthermore, SEM analysis revealed that the expansion of *V. nigrum* had a more pronounced influence on the fungal community diversity than on the bacterial community diversity (Figure 6), which aligns with the findings of Yang et al. (2024). Several mechanisms may explain this discrepancy. Firstly, it has been shown that fungal diversity is more sensitive to grassland degradation than bacterial diversity (Wang et al., 2022). Numerous studies have demonstrated that the correlation between fungal diversity and soil nutrients is stronger in degraded grasslands than in bacterial diversity (Wu et al., 2021; Yang et al., 2017). This phenomenon is attributed to fungal communities being more significantly influenced by substrate quality and heterogeneity than bacterial communities (Li et al., 2019). It is important to note that soil TP content, rather than soil pH, predominantly mediates changes in the soil fungal community diversity after the expansion of *V. nigrum*. A previous study also indicated that the soil P concentration had a significant positive effect on soil fungal abundance (Yan et al., 2022). One possible explanation for this pattern is that soil fungi can enhance phosphorus uptake by plants and facilitate associations between soil fungi and plants, thereby contributing to an increase in the biomass of soil fungi (Zhang et al., 2014).

Furthermore, SEM analysis provided additional evidence that the expansion of *V. nigrum* directly influenced the composition of the fungal community (Figure 7b). This effect may be attributed to allelopathic substances released by *V. nigrum* during expansion, which can alter fungal community composition to some extent. Numerous studies have demonstrated that root exudate metabolites can modify the structure and function of various microorganisms (Zhalnina et al., 2018). Yuan emphasized the significance of organic acids in plant-microbe interactions (Yuan et al., 2015). For instance, the addition of organic acids as the sole substrate significantly enhances the colonization of tomato roots (Lugtenberg et al., 1999). Therefore, the secretion of chemosensory chemicals by *V. nigrum* affects soil microorganisms and fosters an inter-root microbial community conducive to growth, thereby enhancing its competitive ability for expansion. In summary, the expansion of *V. nigrum* directly influenced the composition of fungal communities, while the enhanced nutrient availability indirectly promoted increased microbial community diversity, thereby creating conditions more favorable for its expansion and proliferation. Understanding how microbial communities respond to the expansion of *V. nigrum* and how this response contributes to the colonization of *V. nigrum* is essential for assessing soil nutrient availability and grassland ecology.

## 5 Conclusion

Our study elucidated the effects of *V. nigrum* expansion on the soil physicochemical properties and microbial community structure. We found that expansion of *V. nigrum* decreased soil pH, increased soil nutrient content, and altered bacterial and fungal *β*-diversity. In contrast, soil bacterial and fungal communities exhibited different responses to this expansion, with soil fungal communities demonstrating a greater sensitivity to the expansion of *V. nigrum*. Furthermore, microbial co-occurrence network analyses revealed that the effect of *V. nigrum* expansion on the soil environment led to an increase in competitive microbial interactions at certain levels. SEM provided robust evidence that the expansion of *V. nigrum* modifies soil fungal composition and indicated that changes in soil TP content were the primary drivers of alterations in fungal community diversity. Our findings underscore that soil fungal community composition is directly driven by the dispersal of *V. nigrum*, while fungal community diversity is driven by soil total phosphorus content. Furthermore, the expansion of *V. nigrum* significantly enhanced soil nutrient availability, altered soil microbial communities and their symbiotic networks, and promoted its own colonization and expansion by shaping a favorable soil environment. These findings suggest that *V. nigrum* expansion positively influences nutrient cycling and enhances soil microbial diversity, offering novel insights into the mechanisms by which *V. nigrum* expansion affects soil communities and nutrient characteristics.

## Data Availability

The datasets presented in this study can be found in online repositories. The names of the repository/repositories and accession number(s) can be found in the article/Supplementary material.
