# Supplementary material for: Effects of Veratrum nigrum expansion on soil microbial community structure in inner Mongolian mountain steppe
Source: Front Microbiol. 2025 Oct 28;16:1662400. doi: 10.3389/fmicb.2025.1662400 (PMC12602426; doi:10.3389/fmicb.2025.1662400)
Supplement: Supplementary file 1 [file Table_1.DOCX]

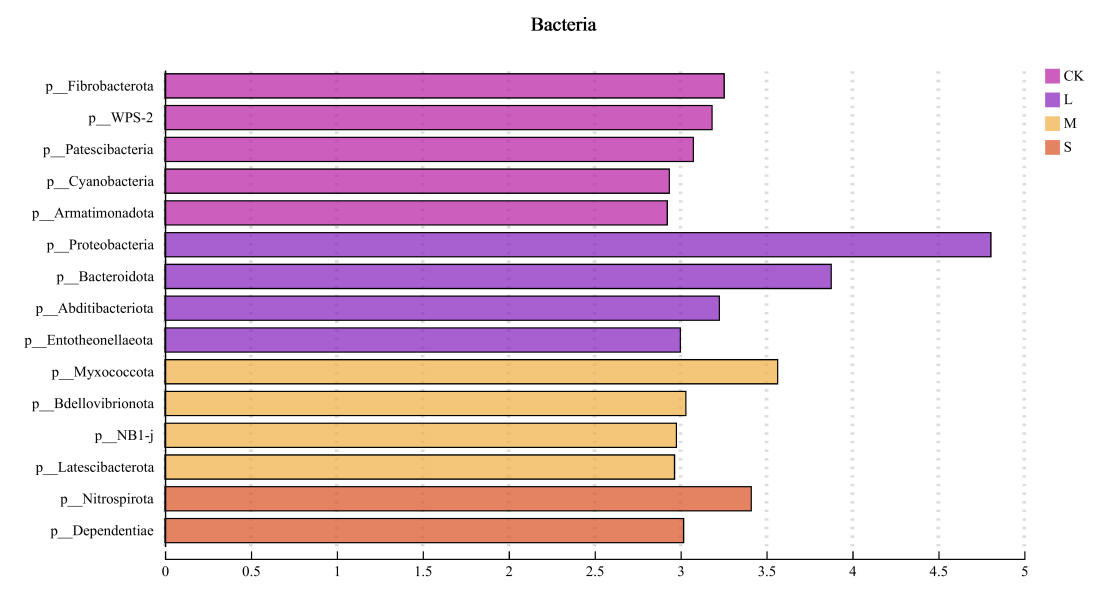


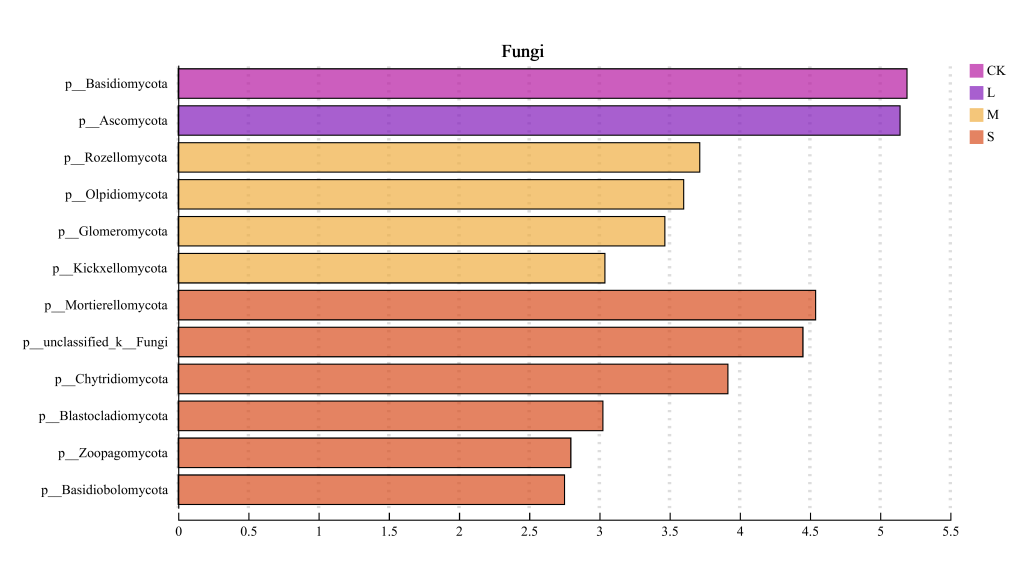


**Fig. S1.** Differential taxa in (a) bacterial and (b) fungal communities calculated by linear discriminant analysis (LDA) effect size (LEfSe).

**Table S1.** Bacterial and fungal network properties at various stages of degradation.

| Network Parameters | Bacterial | | | | Fungal | | | |
| --- | --- | --- | --- | --- | --- | --- | --- | --- |
|  | CK | L | M | S | CK | L | M | S |
| Nodes | 185 | 184 | 176 | 173 | 120 | 156 | 154 | 153 |
| Links | 969 | 1700 | 1621 | 1451 | 409 | 572 | 545 | 613 |
| Positive links | 85.66% | 85.76% | 82.11% | 84.15% | 86.06% | 85.31% | 84.77% | 84.83% |
| Negative links | 14.34% | 14.24% | 17.89% | 15.85% | 13.94% | 14.69% | 15.23% | 15.17% |
| Network diameter | 10 | 11 | 10 | 9 | 10 | 8 | 10 | 9 |
| Modularity | 0.693 | 0.521 | 0.58 | 0.6 | 0.741 | 0.7 | 0.72 | 0.687 |
| Average clustering coeffcient | 0.543 | 0.498 | 0.542 | 0.596 | 0.494 | 0.426 | 0.464 | 0.42 |
| Average path length | 4.162 | 3.672 | 3.694 | 3.771 | 4.492 | 4.098 | 4.275 | 4.064 |
| Average degree | 0.057 | 0.101 | 0.105 | 0.098 | 0.057 | 0.47 | 0.046 | 0.053 |
